# VeriAbs : Verification by Abstraction and Test Generation (Competition Contribution)

**DOI:** 10.1007/978-3-030-45237-7_25

**Published:** 2020-03-13

**Authors:** Mohammad Afzal, Supratik Chakraborty, Avriti Chauhan, Bharti Chimdyalwar, Priyanka Darke, Ashutosh Gupta, Shrawan Kumar, Charles Babu M, Divyesh Unadkat, R Venkatesh

**Affiliations:** 8 Johannes Kepler University, Linz, Austria; 9 University of Birmingham, Birmingham, UK; 10 Tata Research Development and Design Center, Pune, India; 11 Indian Institute of Technology, Bombay, India; 12 Chennai Mathematical Institute, Chennai, India

## Abstract

VeriAbs is a strategy selection based reachability verifier for C code. It analyzes the structure of loops, and intervals of inputs to choose one of the four verification strategies implemented in VeriAbs. In this paper, we present VeriAbs version 1.4 with updates in three strategies. We add an array verification technique called *full-program induction*, and enhance the existing techniques of loop pruning, *k*-path interval analysis, and disjunctive loop summarization. These changes have improved the verification of programs with arrays, and unstructured loops and unstructured control flows.

## 1 Verification Approach

VeriAbs is a reachability checker for C code that employs a portfolio of techniques and works by smartly selecting a sequence of techniques for each problem instance. Specifically, it performs structural and interval analysis of the input code to determine a sequence of suitable verification techniques, or a strategy [2]. An earlier version of the tool appeared in [9]. Figure 1 shows the architecture with this year's enhancements in dashed lines. When the input program contains unstructured loops, VeriAbs performs fuzz testing in parallel with *k*-induction. If the program does not contain unstructured loops but loops manipulating arrays, VeriAbs applies array abstraction techniques like loop shrinking, loop pruning, and full-program induction [7] in sequence. If the program contains inputs of very short ranges, VeriAbs applies explicit state model checking, and loop invariant generation using program behaviour, syntax and counter-examples in parallel [2]. Otherwise VeriAbs applies *k*-path interval analysis, loop abstraction, loop summarization, bounded model checking, and *k*-induction in the order presented in the architecture. If any technique successfully (in)validates the encoded properties, the tool reports the result, generates the witness, and exits. We next explain the enhancements made to VeriAbs this year.

### 1.1 Tool Enhancements

*Full-Program Induction.* VeriAbs applies full-program induction as presented in [7] to programs manipulating arrays of a symbolic size $N$ given as a parameter. It takes as input

⋆ Jury member, corresponding author : priyanka.darke@tcs.com

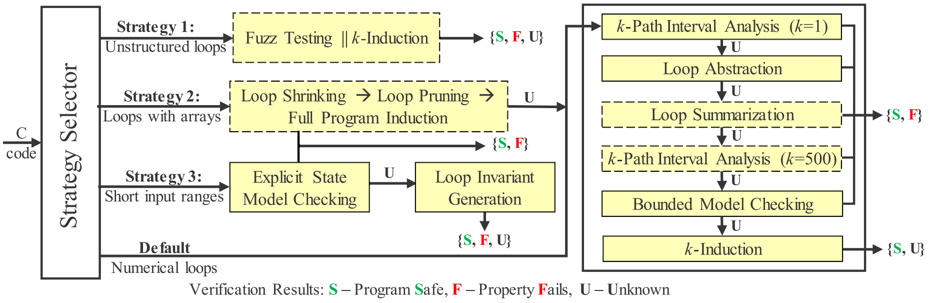

**Fig. 1.** Architecture Diagram a parameterized program represented by $\mathsf{P}_N$, annotated with parameterized pre- and post-conditions represented by $\varphi(N)$ and $\psi(N)$ respectively and checks the validity of the Hoare triple $\{\varphi(N)\}\,\mathsf{P}_N\,\{\psi(N)\}$ for all values of $N$ $(>0)$. We summarize the technique in [7] here.

In the base case, it verifies that the given Hoare triple holds for a fixed number of values of $N$ (say for $N=1$). If the check fails, a property violation is reported. It then hypothesizes that the Hoare triple $\{\varphi(N-1)\}\,\mathsf{P}_{N-1}\,\{\psi(N-1)\}$ holds for $N>1$, where $\mathsf{P}_{N-1}$ is the program with parameter $N-1$. In the induction step, the technique synthesizes a code fragment $\partial\mathsf{P}_N$, called the *difference program*, such that $\{\varphi(N)\}\,\mathsf{P}_N\,\{\psi(N)\}$ is valid iff $\{\varphi(N)\}\,\mathsf{P}_{N-1};\partial\mathsf{P}_N\,\{\psi(N)\}$ is valid. The *difference program* is the computation to be performed after the program $\mathsf{P}_{N-1}$ has executed to get the same state as $\mathsf{P}_N$. It then computes a formula $\partial\varphi(N)$, called the *difference pre-condition*, such that $\varphi(N)$ is implied by the conjunction of $\varphi(N-1)$ and $\partial\varphi(N)$, and that $\partial\varphi(N)$ continues to hold after the execution of $\mathsf{P}_{N-1}$. The induction step now needs to prove the validity of $\{\psi(N-1)\wedge\partial\varphi(N)\}\,\partial\mathsf{P}_N\,\{\psi(N)\}$. It uses weakest pre-condition computation to infer formulas $pre(N)$ over the variables and arrays whose values were computed by $\mathsf{P}_{N-1}$ and subsequently read in $\partial\mathsf{P}_N$. Base case is checked for $pre(N)$ and it is subsequently used to strengthen the pre- and post-conditions in the inductive step. The technique, thus, inducts over the entire program via the parameter $N$, in place of inducting over individual loops by using specialized predicates as in [6]. Full-program induction does not rely on inductive invariants for each loop in the program.

```
1  b=0, d=0, c=30;
2  a = *;
3  if (a == 10)
4      c = 30; //Path P1
5  else if (a < 10)
6      b = 3;  //Path P2
7  else if (a > 10)
8      d = 31; //Path P3
9  if (c==30 && a==10)
10     d = 31;
11 if(a >= 10)
12     assert(d == 31);
```

**Fig. 2.** Example

*k-Path Interval Analysis.* VeriAbs implements a *k*-path interval analysis which is an extension of the standard non-relational interval domain [2]. It maintains the path-wise data ranges of variables along a configurable *k* number of paths at each program point, thus matching the precision of relational domains. When the number of paths at the join point exceeds *k*, a subset of paths are merged to maintain *k* paths at the join point. In previous versions, arbitrary subsets of paths were merged. For SV-COMP 2020, the join operation identifies variables of interest (VOIs) with respect to the given property to decide which paths to merge such that VOIs can retain precise values.

Consider the example shown in Figure 2 with a valid property at line 12 to be analyzed with *k=2* and the VOI `d`. It can be seen that three paths – P1, P2 and P3 join at line number 9. The enhanced join operation merges paths P1 and P2 so that the resultant paths are as follows:

`P1+P2: {a=[MIN,10], b=[0,3], c=30, d=0},`
`P3: {a =[11,MAX], b=0, c=30, d=31}.`
This information at the join point helps validate the property. Earlier, the join operation could merge the path P3 with P1 or P2, leading to an imprecise interval – [0,31] of `d` at the join point, resulting in spurious property violation. Our implementation considers variables used in the encoded property as the VOIs.

*Loop Pruning* is an array abstraction technique that defines a set of criteria (and a resulting set of program transformation rules) which if satisfied by loops processing arrays, it is sufficient to analyze the first few elements instead of the entire array [14]. In this version, pruning has been extended to programs containing nested loops and multidimensional arrays. By structural analysis, we identify if elements of the multidimensional array are processed *uniformly* in loops. If yes, we compute reduced dimensions of the array (for example, `a[m][m]` may be reduced to `a[4][4]`). We have also refined the pruning criteria to improve its applicability over multidimensional and dynamically allocated arrays, 56 additional SV-COMP'20 ReachSafety benchmarks are solved by the current implementation of array pruning as compared to the previous version.

*Disjunctive Loop Summarization.* VeriAbs analyses interleavings of unique paths within a loop to produce its disjunctive summary to find errors and proofs [2]. In the current version, VeriAbs extends this technique in the following situations: (a) while it earlier restricted affine transformations to identity matrices, we now allow diagonal matrices with finite monoid [4]; (b) we use the approach of generating *flattenings* as shown in [4] for loops which are *flattable*; (c) we use VeriAbs' general philosophy of deriving over-approximate summaries using the techniques in [12], when precise disjunctive summary is not derivable.

## 2 Software Architecture

VeriAbs is primarily developed in Java and Perl. It implements all program analyses (except full-program induction) and program transformers in Prism [13], the TCS Research program analysis framework. It transforms programs processing multidimensional or dynamically allocated arrays in loops to equivalent programs with symbolically sized 1D arrays. This transformed program is consumed by VAJRA v1.0 [7], the tool that implements full-program induction. VAJRA uses LLVM v6.0.0 [15] compiler infrastructure for program transformations and Z3 SMT solver v4.8.7 [10] for checking the validity of Hoare triples and for computing weakest pre-conditions. For BMC VeriAbs uses the C Bounded Model Checker (CBMC) v5.10 [8] with the Glucose Syrup SAT solver v4.0 [3]. For fuzz testing we enhance American Fuzzy Lop [16] to allow test case mutation within valid data ranges generated by $k$-path interval analysis for better path coverage. VeriAbs uses $k$-induction with continuously refined invariants as implemented in CPAchecker v1.8 [5] for an improved precision over our existing light weight implementation of $k$-induction.

In this version, we additionally derive disjunctive invariants for correctness witnesses using abstract acceleration and abstract interpretation, and add them to the control flow automaton generated by CPAchecker. If all implemented techniques fail, we use techniques implemented in Ultimate Automizer v3204b741 [11] to generate correctness witnesses.

## 3 Strengths and Weaknesses

The main strengths of VeriAbs are (1) strategy selection that correlates strengths of verification techniques and input code properties, and (2) a portfolio of sound techniques. Weaknesses:

(1) long strategies – the lengths of strategies executed by VeriAbs in the worst case can be ten techniques, thus time consuming. Hence, smarter and shorter strategies are needed. (2) Non-linear expressions in loops – loop abstractions in VeriAbs assign non-deterministic values to variables modified in such expressions. (3) Multidimensional arrays in loops manipulating non-contiguous locations – these are limitations of loop shrinking and pruning. These weaknesses are not limitations of the state-of-the-art, and appropriate techniques if integrated into VeriAbs can be easily invoked by the strategy selector to enable verification of such programs.

## 4 Tool Setup and Configuration

The VeriAbs SV-COMP 2020 executable is available for download at https://gitlab.com/sosy-lab/sv-comp/archives-2019/tree/master/2020/veriabs.zip. To install the tool, download the archive, extract its contents, and then follow the installation instructions in `VeriAbs/INSTALL.txt`. To execute VeriAbs, the user needs to specify the property file of the respective verification category using the `--property-file` option and the `-64` option for programs with a 64 bit architecture. The witness is generated in the current working directory as `witness.graphml`. A sample command is as follows:

```
VeriAbs/scripts/veriabs <-64> --property-file ALL.prp example.c
```

VeriAbs participated in the ReachSafety and the SoftwareSystems-ReachSafety categories of SV-COMP 2020. The BenchExec wrapper script for the tool is `veriabs.py` and the benchmark description file is `veriabs.xml`.

## 5 Software Project and Contributors

VeriAbs is maintained by some members of the Foundations of Computing group at TCS Research [1]. They can be contacted at veriabs.tool@tcs.com. We are thankful to the developers of American Fuzzy Lop, CBMC, CPAchecker, Glucose Syrup, LLVM, UAutomizer and Z3 for allowing us to use the tools within VeriAbs.
